# Evolution of DNA Methylation Patterns in the Brassicaceae is Driven by Differences in Genome Organization

**DOI:** 10.1371/journal.pgen.1004785

**Published:** 2014-11-13

**Authors:** Danelle K. Seymour, Daniel Koenig, Jörg Hagmann, Claude Becker, Detlef Weigel

**Affiliations:** Department of Molecular Biology, Max Planck Institute for Developmental Biology, Tübingen, Germany; University of California Irvine, United States of America

## Abstract

DNA methylation is an ancient molecular modification found in most eukaryotes. In plants, DNA methylation is not only critical for transcriptionally silencing transposons, but can also affect phenotype by altering expression of protein coding genes. The extent of its contribution to phenotypic diversity over evolutionary time is, however, unclear, because of limited stability of epialleles that are not linked to DNA mutations. To dissect the relative contribution of DNA methylation to transposon surveillance and host gene regulation, we leveraged information from three species in the Brassicaceae that vary in genome architecture, *Capsella rubella*, *Arabidopsis lyrata*, and *Arabidopsis thaliana*. We found that the lineage-specific expansion and contraction of transposon and repeat sequences is the main driver of interspecific differences in DNA methylation. The most heavily methylated portions of the genome are thus not conserved at the sequence level. Outside of repeat-associated methylation, there is a surprising degree of conservation in methylation at single nucleotides located in gene bodies. Finally, dynamic DNA methylation is affected more by tissue type than by environmental differences in all species, but these responses are not conserved. The majority of DNA methylation variation between species resides in hypervariable genomic regions, and thus, in the context of macroevolution, is of limited phenotypic consequence.

## Author Summary

DNA methylation is an epigenetic mark that has received a great deal of attention in plants because it can be stably transmitted across generations. However, the rate of DNA methylation change, or epimutation, is greater than that of DNA mutation. In addition, different from DNA sequence, DNA methylation can vary within an individual in response to developmental or environmental cues. Whether altered characters can be passed on to the next generation via directed modifications in DNA methylation is a question of great interest. We have compared how DNA methylation changes between species, tissues, and environments using three closely related crucifers as examples. We found that DNA methylation is different between roots and shoots and changes with temperatures, but that such changes are not conserved across species. Moreover, most of the methylated sites are not conserved between species. This suggests that DNA methylation may respond to immediate fluctuations in the environment, but this response is not retained over long evolutionary periods. Thus, in contrast to transcriptional responses, conserved epigenetic responses at the level of DNA methylation are not widespread. Instead, the patterns of DNA methylation are largely determined by the evolution of genome structure, and responsive loci are likely short-lived accidents of this process.

## Introduction

Cytosine methylation is a heritable epigenetic modification found in the genomes of organisms spanning the eukaryotic phylogeny [1], [2], [3], [4]. It occurs in three nucleotide contexts, CG, CHG, or CHH (where H is any nucleotide except G) [5], and is enriched in the repeat rich heterochromatic regions of genomes, in nucleosome linkers, and at CG sites in the exon sequences of genes (gene body methylation) [4], [6], [7], . Repeat-localized DNA methylation plays a role in transposon silencing [12], [13], but the direct relationship between transcription of protein coding genes and DNA methylation remains unclear. In contrast to repeat methylation, gene body methylation is associated with moderately transcribed sequences [6], [7], [14], [15], [16], and has been proposed to stabilize gene expression levels by excluding H2A.Z [17]. Nevertheless, DNA methylation can vary between tissues and environments [18], [19], [20], and in a handful of cases changes in methylation state contribute to heritable phenotypic variation, although the majority have been linked to structural differences near the affected genes [21], [22], [23], [24], [25], [26], [27]. These observations suggest that DNA methylation may regulate developmental processes and that it could potentiate phenotypic variation during evolution.

Unlike mutational processes acting on DNA sequences, our understanding of the factors contributing to meiotically stable variation in DNA methylation is in its infancy [28]. The different molecular mechanisms governing DNA methylation constitute one factor impacting stability and subsequent inheritance at symmetric and asymmetric sites. In the plant *Arabidopsis thaliana*, initiation and maintenance of methylation at CG and CHG sites is divided primarily between DNA METHYLTRANSFERASE 1 (MET1) and CHROMOMETHYLASE3 (CMT3) [29], [30], [31]. During DNA replication these two enzymes copy symmetrically methylated cytosines onto the newly synthesized DNA strand using the parental strand as a template [32], [33]. Unlike symmetric cytosine methylation, CHH methylation cannot be replicated from the template strand [34]. Instead, methylation at newly synthesized CHH sites is established after cell division by the RdDM RNA-directed DNA methylation pathway through the concerted action of small RNAs (sRNAs) produced from the methylated locus and the de novo DNA methyltransferases DRM1/DRM2 (DOMAINS REARRANGED METHYLTRANSFERASE1/2) [34], [35], [36], [37]. In addition, RdDM-independent asymmetric DNA methylation relies on DDM1 (DECREASE IN DNA METHYLATION1) and CMT2 [38].

The extent to which DNA methylation varies at individual sites across generations, or the epimutation rate, has only recently been characterized in isogenic plant lines [39], [40]. Repeat-associated methylation was remarkably stable over 30 generations, but some variability arose outside of repeats in euchromatic sequence [39], [40]. Changes in DNA methylation accumulated non-linearly, indicating that a subset of methylated sites is particularly prone to spontaneous changes in methylation and, as a result, the absolute DNA methylation differences quickly reach saturation [39], [40]. Variation of methylation across generations has been linked to the transgenerational cycling of transposon and repeats between methylated and unmethylated states in the germline [41].

Armed with the knowledge of within-species epimutation rate, the degree of epigenome stability over short evolutionary periods, within a single species, for example, can be addressed [18]. Using *A. thaliana*, intraspecific variation in methylation was surveyed in 140 geographically diverse accessions [18]. Most single site and RdDM-derived regional epimutations were rare, occurring in only a few of the 140 accessions [18]. The lack of intermediate frequency epimutations in these categories is consistent with the view that the vast majority of new methylation variants within a species may only exist for brief periods during evolution. Not too surprisingly, a significant subset of both rare and intermediate frequency RdDM-derived regional epimutations were associated with previously unknown structural variants [18]. Expansion and contraction of repeat-associated sequences leads to intraspecific structural variation; therefore, as a result of RdDM silencing, such structural variants should be linked to methylation variation.

Over longer evolutionary periods, broad similarities in DNA methylation are observed across a variety of genomic features. Large-scale patterns of methylation are shared across flowering plants, including extensive methylation of heterochromatic transposon and repeat-associated sequences [6], [7], [8], [9], [10], [11] likely due to conservation of the RdDM machinery in plants. Over shorter divergence times, similar levels of gene body methylation have been observed at orthologous genes within the grasses [11], [42]. Similarly, in vertebrates, where most of the CG sites in the genome are methylated, absence of methylation at so-called CpG islands is usually found in all species examined [43]. Regardless of organism, the degree of DNA methylation conservation depends on both the evolutionary time scale under consideration and on the genomic feature of interest.

Here we compare at single base resolution DNA methylation in three closely related Brassicaceae - *Capsella rubella*, *Arabidopsis lyrata*, and *Arabidopsis thaliana*. These three species, which diverged about 10 to 20 million years ago [44], vary in genome size and architecture [45], [46], [47]. Both *C. rubella* and *A. lyrata* have a Brassicaceae typical set of eight chromosomes, while *A. thaliana* has only five chromosomes [48], [49]. Both the *A. lyrata* and *C. rubella* genomes are about 50% larger than that of *A. thaliana*, but for very different reasons. Expansion of centromeric, heterochromatic regions has enlarged the *C. rubella* genome, but predominantly euchromatic regions have expanded in *A. lyrata*, driven by insertions of transposable elements (TEs) adjacent to genic sequences [46], [47]. Reflecting these differences in genome architecture, the reference genome assemblies represent about 85% of the entire genome in *A. lyrata*, about 75% in *A. thaliana*, and about 60% in *C. rubella* (Table S1) [46], [47], [50], [51], [52], [53], [54]. We show that the difference in genome structure is a major factor influencing the evolution of DNA methylation in these species. Furthermore, while overall DNA methylation is similar between species at many sites, dynamic DNA methylation responses between environments and tissues are rarely conserved. Using a comparative framework we were able to disentangle the contribution of genomic, environmental, and developmental factors to DNA methylation variation between species.

## Results

### Genome-wide distribution of DNA methylation

Using a factorial design, we subjected seedlings of the inbred reference strains, *A. thaliana* Col-0, *A. lyrata* MN47, and *C. rubella* MTE, to either a control or 23-hour cold treatment and separately harvested root and shoot tissues. This design provides the opportunity to determine conservation of DNA methylation as well as dynamic changes between and within species. In addition to extracting DNA for bisulfite-sequencing in duplicate, we also extracted RNA in triplicate for RNA-seq.

Bisulfite-treated samples were sequenced to an average of 20× strand-specific coverage (Table S2). With this coverage, over 97.5% of the cytosines in the non-repetitive portion of the reference genome of each species could be interrogated (99.5% for *C. rubella*, 97.5% for *A. lyrata*, and 98.7% for *A. thaliana*). With a minimum coverage of three, we confidently estimated methylation rates at two thirds to three quarter of cytosines (62% for *C. rubella*, 65% for *A. lyrata*, and 75% for *A. thaliana*). Sites with significant methylation levels were identified using a binomial test [39]. False positive rates, determined from incomplete conversion of exogenous unmethylated phage lambda DNA, were very low (Table S3).

**Figure 1:**
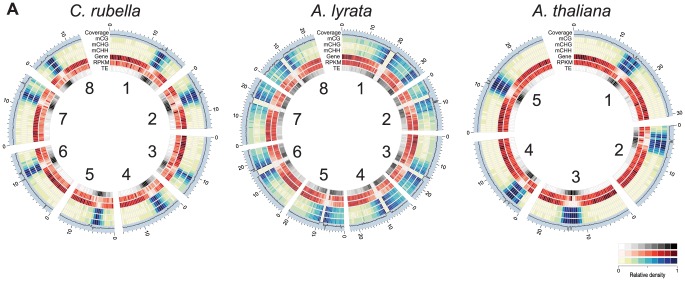
Genomic distribution of DNA methylation. A) Circos plots [74] of *C. rubella*, *A. lyrata*, and *A. thaliana*. Chromosome number is indicated on the inner circle. Data is plotted for 500 kb windows, except for sequencing coverage (100 kb). Gene expression (RPKM) was calculated using the sum of the expression counts from all samples within a species.

**Figure 2:**
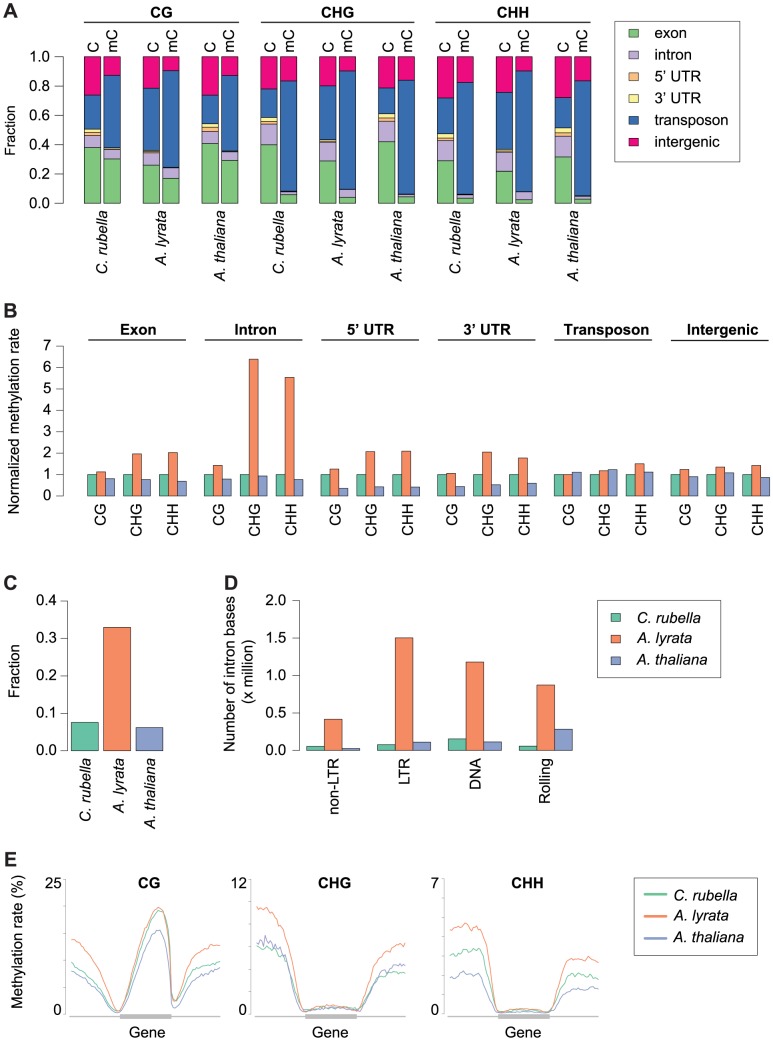
Impact of repeat expansion on DNA methylation at genomic features. A) Feature annotation of all cytosines and methylated cytosines. Annotations are shown for all three contexts. B) Genome average of methylation rates for each genomic feature. Methylation rates are normalized to the outgroup species *C. rubella*. C) Fraction of intron bases annotated as transposable element or other repeat sequence. D) Total number of intron bases (millions) that are annotated as a particular transposable element class. E) Methylation rate distribution across gene bodies of orthologous genes and flanking sequences (1.5 kb up - and downstream). Orthologs that lacked methylation in both their gene body and flanking sequences were excluded. Distributions are plotted by context.

Global patterns of DNA methylation in *A. lyrata* and *C. rubella* are similar to those reported before for *A. thaliana*, with highest levels in regions near the centromeres, which are populated by TEs and repeats, but contain few genes [6], [14], [15] (Fig. 1). There is little correlation between DNA methylation density and gene expression at the 500 kb scale (Fig. 1). Centromeric regions are plagued with TEs, and as expected, methylation is found preferentially at sites annotated as residing in TEs (Fig. 2A). Methylation at CHG and CHH sites, which account for over half of methylated sites in all three species, occurs almost exclusively in TEs (Fig. 2A).

Methylation patterns in the three species reflect their genome architecture. While we mapped a similar number of methylated cytosines in *A. thaliana* and *C. rubella*, consistent with the almost equal size of euchromatic sequences in both species, we identified almost three times as many methylated cytosines in *A. lyrata*, even though its reference genome assembly is only 50 to 75% longer than that of the other two species. The larger number of methylated cytosines in *A. lyrata* has led to an elevation in the methylation rate at a number of genomic features (Fig. 2B). This increase has only occurred at CHG and CHH sites, hallmarks of RdDM at TEs, and is especially evident in introns, correlating with the invasion of introns by TEs in this species (Fig. 2B, C). Almost one third of intronic bases in *A. lyrata* overlap with a TE or repeat, compared to fewer than 10% in the other two species (Fig. 2C), with the expansion found for all TE classes (Fig. 2D). Intron-inserted TEs are frequently found in non-expressed genes (Fig. S1) and are associated with increased methylation in flanking intronic and exonic sequences (Fig. S2), potentially due to pseudogenization or incomplete annotation of repeats. However, when a TE is inserted into the intron of an expressed gene, elevation of CHG and CHH methylation of exon sequences is not evident (Fig. S2, S3). Despite TE expansion in *A. lyrata*, the level of *A. lyrata* gene body methylation is comparable to that of *C. rubella*, which has few TEs in its introns (Fig. 2E). However, species-specific differences in methylation patterns are evident in flanking UTR and intergenic sequence (Fig. 2E). In these regions *A. lyrata* is the most highly methylated in all contexts (Fig. 2E). Depending on context, *C. rubella* displays methylation levels either similar to *A. thaliana* or intermediate between the two other species (Fig. 2E).

**Figure 3:**
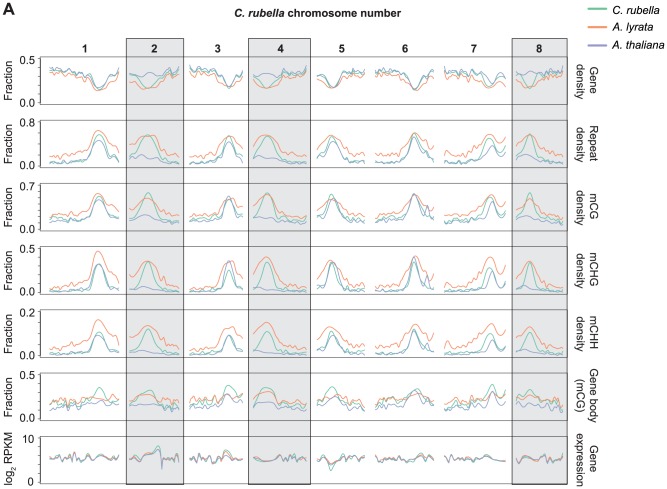
Centromere loss impacts DNA methylation in *A. thaliana*. A) Orthologous genes, anchored on the *C. rubella* genome, were used to calculate several statistics to investigate the impact of centromere loss on DNA methylation in *A. thaliana*. *Capsella rubella* centromeres 2, 4, and 8 (grey boxes) were lost during chromosomal fusion events that occurred on the branch leading to *A. thaliana*. Gene density, repeat density, and methylation densities were calculated for a 20 Kb window centered on the midpoint of each orthologous gene (10 kb up- and 10 kb downstream). Gene density and repeat density were calculated as fractions of each 20 kb window annotated as either a gene (ATG to STOP) or a repeat. Methylation densities were calculated as fractions of cytosines methylated in each context. Gene body methylation and gene expression (RPKM) were calculated for each ortholog. Gene body methylation was calculated as the fraction of methylated CG sites in a gene (ATG to STOP). Gene expression data from all samples within a species were used to calculate the RPKM values. For each statistic, local linear regression was performed to smooth the data in 250 kb bins. Smoothing parameter was relative to chromosome length.

*Arabidopsis thaliana* lost three centromeres relative to *A. lyrata* and *C. rubella*, and this loss has been estimated to account for about 10% of the genome size reduction in *A. thaliana* [46]. Using orthologous genes, it is possible to reconstruct the gene, repeat, and methylation density using the ancestral chromosome positions (Fig. 3). As expected, repeat density and cytosine methylation next to these degraded centromeres is reduced in *A. thaliana*, while gene density is higher (Fig. 3). Particularly notable is the decrease in CG gene body methylation (Fig. 3). Although gene body methylation is positively correlated with gene expression in several species [6], [7], [14], [15], [16], gene expression is not noticeably different in these regions between the three species (Fig. 3). Thus, the elimination of centromeres has had a measurable impact on repeat and methylation distribution in *A. thaliana*, but did not strongly affect the expression of ancestrally pericentromeric genes.

### Methylated regions are not conserved across species

**Figure 4:**
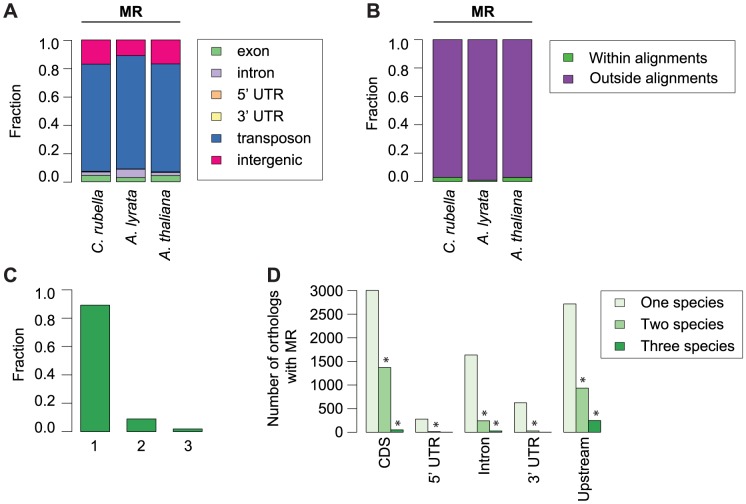
Conservation of methylated regions (MR). A) Annotation of all bases in MRs. B) Fraction of bases in MRs that occur either within or outside of the three-way whole genome alignments. C) Fraction of MR bases found within three-way whole genome alignments that occur in one, two, or three species. D) Conservation of MRs in the absence of sequence alignments. The total number of orthologous genes overlapping an MR in one, two, or three species is given, with location of MR overlap separated by genomic feature. Upstream region was defined as 1 kb before the start codon. Asterisk indicates two or three-way sharing of MRs that exceeds permutation values.

Methylation of plant genomes is driven to a large extent by TEs, which are silenced via either the sRNA-mediated RdDM pathway [36] or the RdDM-independent pathway which relies on DDM1 [38]. Using a Hidden Markov Model algorithm, we identified methylated regions (MR) in each genome, which have a median length of 300 to 530 bp and cover between 26 and 73 Mb (Table S4). MRs are preferentially found in heterochromatic sequence next to centromeres, as they are enriched for TEs (Fig. S4, Fig. 4A). Since TEs are rapidly turned over, we expected MRs to be only poorly conserved. To test this assumption, we identified nearly 60 Mb of sequences with a 1∶1∶1 relationship in whole-genome alignments (Table S5) [47]. Less than 1% of the MR space is contained in the alignable portion of the genomes (Fig. 4B). In the rare cases where an MR spans alignable sequences, such sequences are almost always methylated in only one of the three species (Fig. 4C). We conclude that DNA methylation targets primarily the variable portion of the genome, which is subject to species-specific expansion and contraction of TEs.

To determine whether specific orthologs tend to be associated with methylation in all species, even in the absence of MR sequence conservation, we analyzed orthologs that contained a MR overlapping or within 1 kb of their coding region. Again, we found that the presence of MRs is rarely conserved (Fig. 4D, Table S6), although MR sharing is seen more often than expected by chance (Fig. 4D, Tables S7, S8). This could, however, be simply due to genes near centromeres being more often associated with MRs because they are in an MR-rich genome environment.

### Conservation of CG gene body methylation

**Figure 5:**
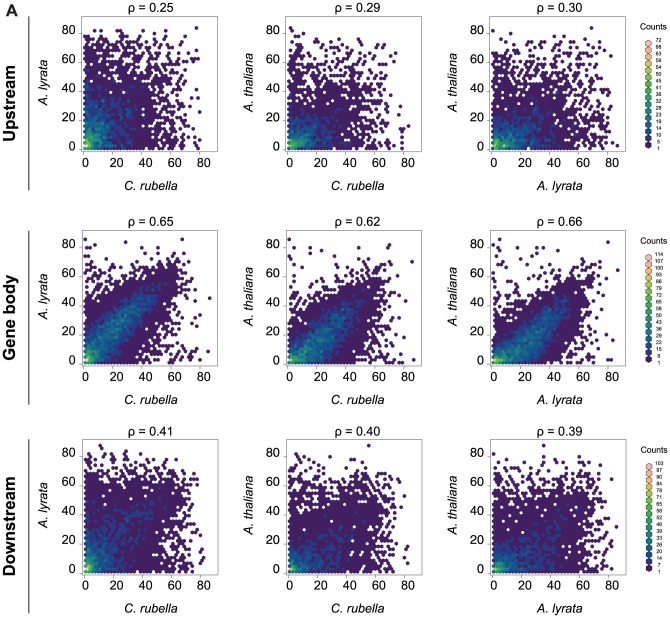
Methylation rates at orthologs. A) Pairwise comparison of the average methylation rates at orthologs. Average methylation rate was calculated as the average of all CG sites in the feature, including non-methylated CG sites. Pairwise comparisons are shown for upstream regions (1.5 kb), gene bodies, and downstream regions (1.5 kb). Spearman rank correlation coefficient (ρ) is included for each comparison.

In contrast to RdDM of TEs and other repeats, the function of CG gene body methylation is still enigmatic, although it correlates positively with gene expression and negatively with mean normalized expression variance, or the coefficient of variation, across tissues and treatments (Fig. S5) [6], [7], [14], [15], [16], [17]. CG gene body methylation is found in the majority of genes (Table S9), and its rate is highly correlated between orthologs, while CG methylation up- and downstream of genes is much less correlated (Fig. 5).

CHG and CHH methylation in gene bodies is often indicative of transcriptionally inactive pseudogenes, paralogs, or transposons wrongly annotated as protein coding genes [14], [15], [55]. Between 10 and 20% of genes exhibit CHG or CHH methylation, most of which were not expressed in our samples (Table S9). Genes with CHG or CHH methylation are underrepresented in the orthologous gene set, where their fraction drops to less than half of their fraction among all genes, supporting the assertion that CHG and CHH methylation point to a tendency toward pseudogenization (Table S9). Moreover, CHG and CHH methylation are generally not conserved, suggesting that these marks arise in a lineage-specific fashion.

### Site-specific gains and losses of methylation in euchromatic sequence

**Figure 6:**
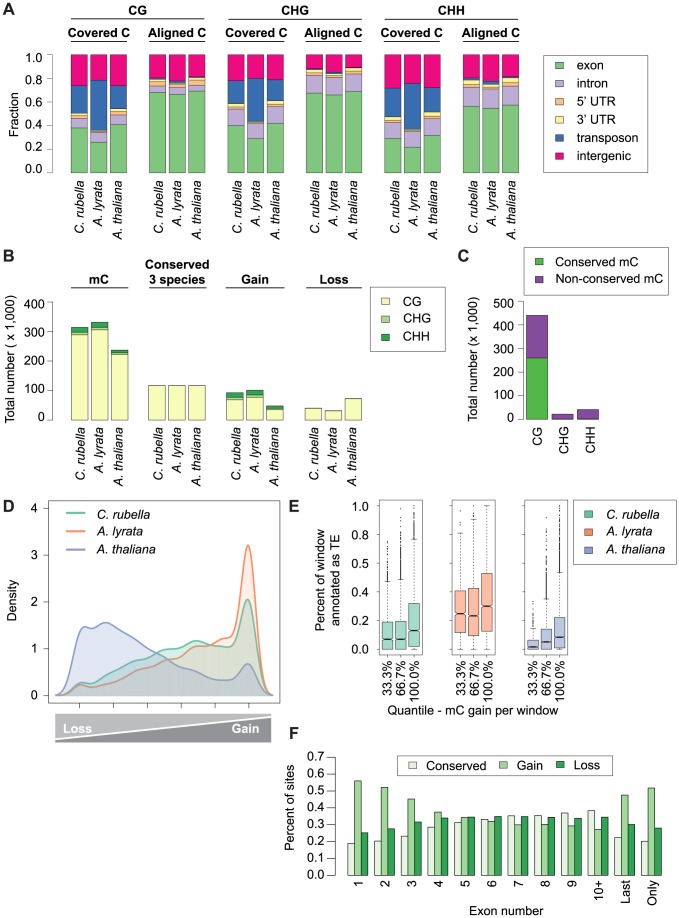
Site-level comparison of methylation. A) Annotation of all cytosines within a species (covered C) compared to the annotation of cytosines found in the three-way whole genome alignments (aligned C). B) Total number of mC by context for aligned site classes. Site classes are as follows: mC - methylated sites within a species. Conserved (3 species) - sites that are methylated in all three species. Gain - sites that are methylated in a single species. Loss - sites that have lost methylation in a single species. C) Total number of conserved mC and non-conserved mC by context. D) Density plot describing the distribution of variable sites in the genome (10 kb windows). For each window the following statistic was calculated: species-specific methylation gains/sum of species-specific methylation gains and losses. E) Windows with a high density of gains have more transposons and repetitive sequences. Density of transposons plotted against density of methylation gains (10 kb window). F) Methylation gains are enriched at the beginning and end of genes. Fraction of mC in each site class is plotted by exon position in a gene.

We used the cross-species alignments to identify 15.1 million conserved CG, CHG and CHH sites, which are located particularly in exons (Fig. 6A, Table S5). Although only a small portion, 2%, had significant methylation, most were shared between at least two species, with *A. thaliana* having the fewest methylated sites, reflecting the general decrease in global DNA methylation in this species (Fig. 6B–D, Table S10). Sites methylated in multiple species are further enriched in exons, with very few of these conserved sites being CHG or CHH sites (Fig. 6B,C, Fig. S6).

Sites that differ in methylation between species can be used to study gain and loss of methylation. We consider sites that are methylated only in a single species as lineage-specific gains, and absence of methylation in only one species as lineage-specific losses. We found that the number of gains and losses reflect the differences in genome architecture between the three species (Fig. 6 B,D). The many methylation losses in *A. thaliana* appear to be the result of genome shrinkage, and this species has also the fewest gains. In contrast, *A. lyrata* has the most gains, likely reflecting recent TE expansion (Fig. 6 B,D). The density of variable sites across the genome (in 10 kb windows) illustrates that gains and losses are not randomly distributed (Fig. 6D). Species-specific gains, which occur in all three sequence contexts, are concentrated in a subset of windows that are strongly enriched for TEs (Fig. 6D,E), but are also frequently found in exons (Fig. S6). That methylation gains are particularly likely in first and last exons suggests that methylation spreading from nearby TEs makes an important contribution to newly methylated sites, regardless of TE class (Fig. 6F, S7) [56], [57], [58].

Lineage-specific losses are more evenly distributed, without any signature of TE association. In addition, sites that are conserved in not only two, but all three species occur across a similar spectrum of genomic features (Fig. S6). Together these results indicate that unlike gains, losses occur in a random fashion, with the proviso that there is an overall global loss of methylation in *A. thaliana* (Fig. 6D). Though centromere elimination contributes to the different methylation pattern in *A. thaliana*, this explains only a minority of these losses (Fig. S8). It appears more likely that they are caused by the global reduction in TE content. We also attempted to understand what factors might contribute to conservation of DNA methylation over time. Sites found in more than one species are enriched in exons of conserved length and are more frequent in the center of exons (Fig. S9, S10).

### Methylation variation within individuals

**Figure 7:**
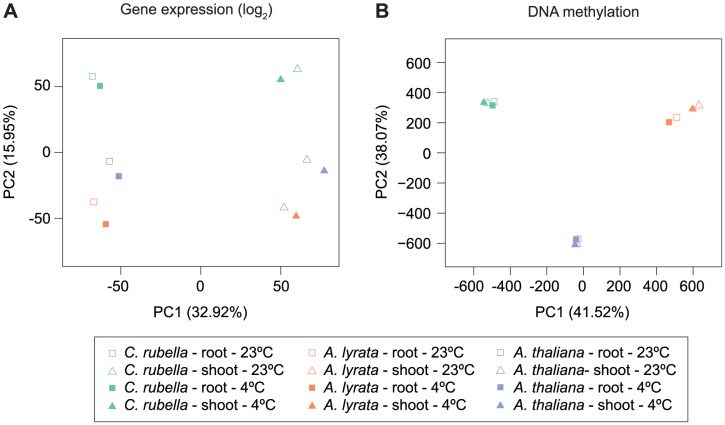
Species gene expression and mC relationships. A) Principal component analysis on fitted gene expression values (log_2_) and B) mC rates at aligned methylated positions. All contexts are considered (see Fig. 6B,C and Table S10 for further description of mC sites).

Because several studies have shown that DNA methylation can change between tissues and in response to external stimuli [19], [20], we wanted to address whether these responses are conserved. Principal component analysis on the four types of samples, control shoots, cold-treated shoots, control roots and cold-treated roots, for all three species according to global RNA-seq measurements revealed that tissue is the most important factor, with over 7,000 genes being differentially expressed between roots and shoots (Fig. 7A, S11). Tissue-specific differences in gene expression are the largest source of expression variance in this data set (Fig. 7A). In contrast, species is the most important factor for differences in DNA methylation and explains 80% of the variance in our data (Fig. 7B, Fig. S12). Moreover, PC2 places *A. lyrata* closest to *C. rubella* instead of its congener *A. thaliana*, reflecting the methylation losses in *A. thaliana* (Fig. 7B).

**Figure 8:**
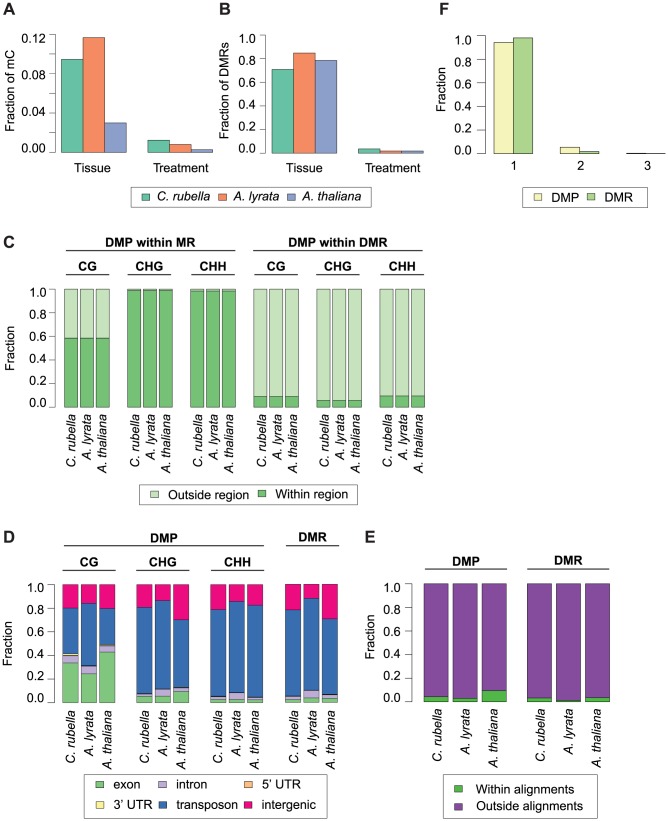
Intraspecific variation in DNA methylation. A) Fraction of mC that are variable between either tissue (root and shoot) or treatment (23°C and 4°C) comparisons. B) Fraction of DMRs that are variable between either tissue (root and shoot) or treatment (23°C and 4°C) comparisons. C) Fraction of DMPs in each context that reside either within a MR or DMR. D) Feature annotation of DMPs by context and DMR bases. E) Fraction of DMPs and DMR bases found within three-way whole genome alignments. F) Fraction of DMPs and DMR bases found within three-way whole genome alignments that occur in one, two, or three species.

To evaluate the degree to which within-species DNA methylation changes are conserved, we first estimated significant differential methylation at site and region levels. Four biologically appropriate comparisons were performed for each species to minimize multiple testing problems. Two tests identified differentially methylated positions (DMPs) between roots and shoots, and two tests identified DMPs between cold and control conditions regardless of tissue type. In each species, ten times as many DMPs were found between tissues than between treatments (Figure 8A, Table S11). Similar to DMPs, 20 to 50 times as many differentially methylated regions (DMRs) were detected between tissues than between treatments (Fig. 8B, Table S12).

Importantly, DMPs and DMRs do not necessarily coincide (Fig. S4, S13). DMPs in all contexts are rarely found within DMRs, indicating that significant regional changes in methylation are not just the extension of single base differences (Fig. 8C). CHG and CHH DMPs reside mainly within MRs (Fig. 8C); since these are almost exclusively found in the non-alignable portions of the genome, including TEs (Fig. 4A, Fig. 8D), the positions of DMPs and DMRs are typically not conserved between species (Fig. 8E). In the rare case that DMPs or DMRs can be found in the portion of a species' genome that can be aligned with the genomes of the other two species (Fig. 8E), they are only variable in a single species (Fig. 8F). Methylation variation at both the site and region level is therefore not conserved across species.

In the absence of sequence conservation at DMRs, we looked for conservation of their presence at orthologous genes. When only considering orthologs, fewer than 700 genes coincide with a DMR (405 in *C. rubella*, 652 in *A. lyrata*, and 221 in *A. thaliana*) (Table S13). Orthologs only rarely shared the presence of an overlapping or adjacent DMR, similar to what we see for MRs. Despite the rarity of such cases, they occur more often than expected by chance for a subset of genomic features and species comparisons (Fig. S14, Table S14, Table S15). Lack of sequence conservation together with minimal overlap of DMR presence at orthologs supports the transitory nature of methylation variation during genome evolution.

We also asked whether differential methylation in or near coding sequences is correlated with changes in gene expression. DMP and DMR overlap with genes was analyzed separately for those that overlapped with exons, introns, 5′ UTRs, 3′ UTRs and 1 kb upstream regions (Table S13, S16). DMPs occur in many genes in all three species, and most of them are expressed in our samples (9,631 in *C. rubella*, 12,216 in *A. lyrata*, and 6,345 in *A. thaliana*), but there is no evidence for correlation between DMPs and gene expression. This holds true for tissue as well as treatment DMPs (average Spearman rank correlation coefficient tissue = −0.04, treatment = 0.02, Table S17). Only a small number of DMRs overlap with expressed genes (529 in *C. rubella*, 801 in *A. lyrata*, and 284 in *A. thaliana*). Again, there is no correlation with gene expression (average Spearman rank correlation coefficient for CG DMRs = −0.16, CHG DMRs = −0.06, CHH DMRs = 0.00, Table S18).

**Figure 9:**
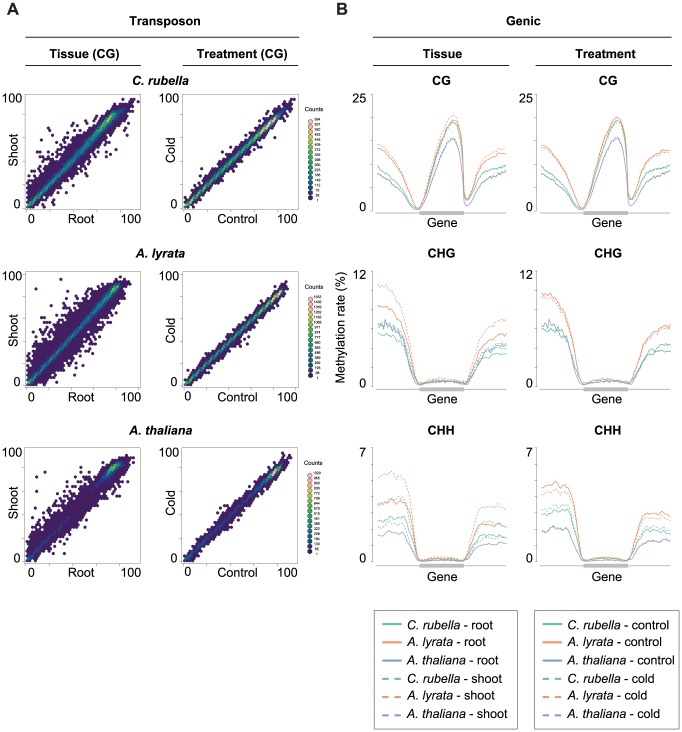
Intraspecific variation of transposon and gene body methylation. A) Comparison of the average methylation rates at annotated transposons and repeats between tissues (root and shoot) and treatments (23°C and 4°C). Average methylation rate is calculated as the average of methylation rates at all cytosines in the feature, including non-methylated cytosines. B) Methylation rate distribution across gene bodies of orthologous genes and flanking sequences (1.5 kb up- and 1.5 kb downstream). Orthologs that lacked methylation in both their gene body and flanking sequences are excluded. Distributions are plotted by context.

Although DMPs and DMRs are not conserved across species, there is consistently more variability between root and shoot samples at a number of genomic features. Importantly, the methylation profile across transposons is quite different between tissues. Transposons are consistently more highly methylated in all sequence contexts in shoots (Fig. 9A). A similar trend is apparent for CHG and CHH sites in intergenic regions in *A. lyrata*, reflecting that TEs are closer to genes in this species (Fig. 9B) [46].

## Discussion

DNA methylation is an ancient epigenetic modification that appears in the genomes of organisms throughout the eukaryotic phylogeny [1], [2], [3]. This mark is associated with a number of cellular processes including transposon silencing and host gene regulation, but the cause-and-effect relationship between gene expression and DNA methylation remains unclear [6], [7], [12], [13], [14], [15], [16]. From an evolutionary standpoint, it is useful to consider methylated cytosines from two differing perspectives, either as a non-canonical nucleotide or as a molecular phenotype akin to transcription, and each perspective has important implications for the interpretation of its evolutionary dynamics.

### Dynamics of DNA methylation as a molecular phenotype

As a molecular phenotype, many characteristics of DNA methylation are conserved between the species we examined. DNA methylation is generally associated with the repeat-dense sequences found in the centromeres, with CG methylation being in addition present at high levels in exonic sequences [6], [7], [8], [9], [10], [11], [14], [15], [16]. Furthermore, gene body methylation levels are conserved in orthologous genes indicating that DNA methylation rate may be subject to purifying selection, a finding consistent with previous wider evolutionary comparisons [42]. The close relationship of the species used in our experiments allows us to make inferences at base pair resolution. Given the substantial rate of epimutation in non-repetitive sequences [39], [40], we were surprised to discover that a large fraction of sites is methylated in more than one species. These sites were predominantly found in gene bodies, providing additional evidence for selective constraint. While gene body methylation is poorly understood, there is some evidence that it is correlated with nucleosome positioning in exons [14], [59]. If nucleosome position is conserved, it could potentially explain long-term conservation of DNA methylation at some sites.

An additional proposed feature of DNA methylation as a molecular phenotype is the ability to respond to external stimuli or internal developmental cues. In theory, such variation could control changes in gene expression. We found evidence for DNA methylation variation in all three species across both tissue type and environment. The changes in DNA methylation were in all three species much greater between tissues, and consistently resulted in lower methylation levels in the root [19]. Differences between the root and shoot tissues also explain a majority of the expression variation in the transcriptional data, but these changes are not directional. We found no evidence that changes in DNA methylation across tissues is associated with changes in gene expression. In fact, a large proportion of methylation changes were found in repetitive sequences. This pattern may result from the increased stringency of transposon silencing in the shoot, which includes the plant germline [60].

While transcriptional responses are highly conserved across all three species, we found no evidence for conservation of DNA methylation response at the sequence level. MRs and DMRs are predominantly found in the rapidly evolving repeat-rich regions of the genome and rarely reside in or near the same orthologous gene in more than one species. In many of the classical epimutants, epigenetic regulation of nearby transposon insertions can impact neighboring genes and cause phenotypic variation [21], [24], [25], [26]. This additional regulation is in some cases beneficial; for example, for genes specifically expressed in the pollen [41], [61]. The data presented here demonstrates that these events are both rare and likely lineage-specific. It is possible that the reported cases of differential methylation as a regulator of transcription are short-term innovations that are eventually replaced by genetically encoded regulation.

### DNA methylation from an epimutational perspective

The mode of inheritance of symmetrically methylated cytosines motivates the interpretation of DNA methylation as a molecular modification that increases the complexity of the genetic code. While mutational processes affecting DNA sequence are well described, epimutational processes are poorly understood. DNA mutations rarely revert and occur in a largely random fashion throughout the genome [62]. In contrast, recent studies have shown that the transgenerational stability of DNA methylation is very context dependent [39], [40]. Over short evolutionary times, epimutations are more likely to occur in euchromatic sequences and are biased away from heavily methylated repetitive sequences [39], [40].

Over the longer evolutionary times examined here, we find that changes in genome content and structure are the major contributors to DNA methylation variation. While the majority of single site and regional methylation is found in repetitive sequences that are unlikely under evolutionary constraint, the remaining observed patterns in euchromatic sequence reflect lineage-specific evolution of transposons. This is particularly obvious in *A. lyrata*, which has experienced a recent invasion of transposable elements into euchromatic sequences [46] and subsequent elevation in the methylation rate of euchromatic features, particularly introns.

Large-scale structural changes that have perturbed the genome-wide DNA methylation landscape have also occurred in *A. thaliana* [48], [49]. Loss of three repeat-rich centromeres in *A. thaliana* caused a decrease in DNA methylation in sequences flanking the ancestral centromeres. The impact of lineage-specific transposon evolution and subsequent methylation is similarly evident in genic sequences. Approximately 40% of methylation in conserved exon sequence is species-specific. These sites are non-uniformly distributed near the 5′ or 3′ edges of genes, likely due to spreading from adjacent transposons [56], [57], [58]. These observations support the hypothesis that surveillance of transposons is the primary contributor to the genomic distribution of DNA methylation in plants. Since transposon content and genome structure vary extensively even over short evolutionary time periods, DNA methylation appears to be similarly variable. This is supported by the poor resolution of species relationships in a principal component analysis of DNA methylation and a nearly ten-fold increase in divergence between *A. lyrata* and *A. thaliana* when comparing DNA methylation as opposed to nucleotide sequence [46]. Together, these results indicate that DNA methylation as a non-canonical nucleotide is very rarely conserved over intermediate evolutionary times scales.

Despite the fact that we can estimate the epimutation rate of methylated cytosines and other parameters related to nucleotide mutations, it is misleading to equate DNA methylation changes to nucleotide substitutions. Our results indicate that the rapid evolution of repeat sequences is the major contributor to the equally rapid changes in the genomic distribution of DNA methylation. In this respect, it is more reasonable to regard DNA methylation primarily as a molecular phenotype resulting from the underlying genetic sequences. Although a few “pure” epialleles have been identified in nature, the majority of natural epimutations are linked to nearby transposon insertions or other genetic changes [21], [24], [25], [26]. Fast evolution of repeat-sequences can, however, provide opportunities for lineage-specific cooption of DNA methylation for regulation of endogenous genes in response to various stimuli.

## Materials and Methods

### Experimental design

Seeds from the reference strain for each species (*A. thaliana* Col-0, *A. lyrata* MN47, *C. rubella* MTE) were sterilized with a 15 minute treatment of 30% bleach and 0.1% Triton X-100. Sterilized seeds were plated onto 0.5× MS 0.7% agar plates with 1% sucrose. Each plate represented a single replicate consisting of 20 seedlings. In total, 7 replicates were sown and randomized into a 3×2×2 factorial design. The three factors in this experiment were species, tissue, and cold treatment. After sowing, plates were stratified in the dark at 4°C for 8 days, before being shifted to 23°C short-day conditions (8 hr light∶16 hr dark). Plates were oriented vertically. After 6 days in 23°C, half of the plates were exposed to 4°C short-day conditions for 23 hours. At the end of the cold treatment, both control (23°C) and treated (4°C) samples were harvested. Root and shoot tissues were harvested independently. Plants were cut just above and below the root-shoot junction to separate the tissues and avoid cross contamination of tissue types. To minimize daily collection times, replicates were blocked by day.

### RNA extraction and RNA-seq library preparation

Total RNA was isolated from three replicates of each factor combination using the Qiagen RNAeasy Plant Mini Kit (catalog # 74904). An on-column DNase digestion was included (catalog # 79254). Total RNA integrity was confirmed on the Agilent BioAnalyzer. Illumina TruSeq RNA libraries were constructed using 3 µg of total RNA. Samples were randomized before library construction. The manufacturer's protocol was followed with one exception - 12 PCR cycles were used instead of the recommended 15. Libraries were quantified on an Agilent BioAnalyzer (DNA 1000 chip). Samples were normalized to 10 nM library molecules and then pooled for sequencing. Three pools were constructed, each consisting of 12 random samples. Each pool was sequenced across three lanes of an Illumina GAII flowcell.

### DNA extraction and bisulfite library preparation

DNA was extracted from two replicates of each factor combination using the Qiagen DNeasy Plant Mini Kit (catalog # 69104). DNA was quantified using the Qubit BR assay (Life Technologies, catalog # Q32853). Bisulfite libraries were confstructed using modifications to the Illumina TruSeq DNA kit and published bisulfite library protocols [15], [39]. Depending on the sample, starting material ranged from 200 ng to 1 µg. Changes to the manufacturer's protocol will be noted here. After shearing of genomic DNA with a Covaris S220 instrument, sheared lambda DNA was spiked into each sample (1∶0.001 sample∶lambda ratio) as a control., for accurate estimation of failure to bisulfite convert non-methylated cytosines. Samples were randomized before library construction. During the ligation step, the amount of adapter was adjusted based on the amount of starting material in each sample. For 1 µg of input DNA, 2.5 µl of adapter were used. Adapter input was scaled linearly for samples with less starting DNA. For the second AMPure bead clean up after the ligation step, the ratio of sample to beads was adjusted to 1∶0.74. A final elution volume of 42.5 µl was used for this step. After ligation, 40 µl of eluate was transferred to a new tube for subsequent bisulfite treatment.

The Qiagen Epitect Plus Kit (catalog # 59124) was used for bisulfite treatment. The manufacturer's protocol for ‘low concentrated and fragmented samples’ was followed, using 85 µl of bisulfite mix for conversion. Clean up of the bisulfite reaction included ethanol as a final wash step. The sample was eluted in 17 µl. After bisulfite treatment samples were amplified using Pfu Cx HotStart Polymerase from Agilent (catalog # 600410) instead of the supplied PCR mix. Reaction conditions are all follows: 32.9 µl of water, 5 µl of 10× Pfu Cx Buffer, 5 µl of 2 mM dNTP, 1.6 µl of Illumina PCR Primer Cocktail, 0.5 µl of Cx Polymerase (2.5 U/µl), 5 µl of bisulfite-treated DNA eluate. Three PCR reactions were pooled for each bisulfite-treated sample. The following cycling conditions were used: 98°C - 30 seconds; 18 cycles of 98°C - 10 seconds, 65°C - 30 seconds, 72°C - 30 seconds; 72°C - 5 minutes. An AMPure bead clean up was used to purify the final PCR product (1∶1 sample to bead ratio). Samples were eluted in 32.5 µl of Illumina supplied Resuspension buffer. 30 µl of the final eluate was transferred to a new plate for subsequent quantification and sequencing. Libraries were quantified using the Agilent BioAnalyzer (DNA 1000 chip). Libraries were diluted to 10 nM and then pooled. Samples were pooled based on genome size - and each pool consists of 2 random samples from each species. Four pools were constructed and each was sequenced across three lanes of the Illumina HiSeq 2000.

### Bisulfite sequencing

We sequenced bisulfite-converted libraries with 2×101 base pair paired-end reads on an Illumina HiSeq 2000 instrument with conventional *A. thaliana* DNA genomic libraries in control lanes. Each sample contained 0.1% lambda DNA as an unmethylated control. We pooled six different samples in each lane. The Illumina RTA software (version 1.13.48) performed image analysis and base calling.

### Processing and alignment of bisulfite-treated reads

Reads were filtered and trimmed as previously described [39]. Subsequently, trimmed reads were mapped against the corresponding reference genomes (Crubella_183, Alyrata_107, Athaliana_167 (TAIR9) [46], [47], [50], [51]. The lambda genome sequence was appended to each species genome sequence in order to estimate the false methylation rates of each sample. All reads were aligned using the mapping tool bismark v0.7.3 [63]. Applying the ‘scoring matrix approach’ of SHORE as previously described [39], we retrieved unique and non-duplicated read counts per position. Read and alignment statistics can be found in Table S2. All command line arguments are listed in Text S1. Raw reads are deposited at the European Nucleotide Archive under accession number PRJEB6701.

### Determination of methylated sites

We used published methods [39], with a few exceptions. Here we retrieved incomplete bisulfite conversion rates, or false methylation rates (FMRs), from the alignments against the lambda genome rather than the chloroplast sequence. False methylation rates are found in Table S3. In addition, we combined the read counts of replicate samples after removing sites that were differentially methylated between replicates. The methylation rates for combined replicates were used for all subsequent analyses. The number of DMPs detected between replicates can be found in Table S19. In each species we required a methylation rate of at least 20% in one of the four tissue-treatment combinations in order for a site to be considered significantly methylated.

### Identification of differentially methylated positions (DMPs)

To identify DMPs we followed published methods [39], but we required positions to have a methylation rate of at least 20% in one of the treatment combinations before performing Fisher's exact test. This increased statistical power by reducing the number of multiple testing corrections. Pairwise tests were not performed between all treatment combinations, instead only relevant comparisons were performed within each species (Root-23°C vs Shoot-23°C, Root-4°C vs Shoot-4°C, Root-23°C vs Root-4°C, Shoot-23°C vs Shoot-4°C).

### Identification of methylated regions (MRs)

To detect contiguously methylated parts of the genome we modified a Hidden Markov Model (HMM) implementation [64]. Briefly, each cytosine can be in either an unmethylated or methylated state. The model trains methylation rate distributions for each state and sequence context (CG, CHG, CHH) independently using genome-wide data. In addition, transition probabilities between the states are trained. To make the original HMM implementation applicable to plant data, three different (beta binomial) distributions were estimated for each state (methylated and unmethylated) instead of just the single distribution used in mammals, which have almost only CG methylation [64]. To prevent identification of regions over uncovered bases, the genome was split at locations that lacked a covered cytosine position for 50 adjacent base pairs. On each of these segments, the most probable path through the methylation states was estimated after genome-wide parameter training. Transitions between states demarcated the methylated regions (MR). Replicates of each treatment combination were combined for this analysis. The combined read counts at cytosines were used to calculate methylation rates, train the HMM, and identify methylated regions. As a result, there is a single segmentation of the genome per treatment combination. Methylated regions were trimmed on both 5′ and 3′ ends by removing positions with a methylation rate below 10%. Further details will be described in a manuscript by Hagmann, Becker et al. [65].

### Identification of differentially methylated regions (DMRs)

Based on the MRs identified for each sample using the HMM algorithm described above, we selected regions of variable methylation state between samples to test for differential methylation. Due to the very large number of MRs, it was critical to reduce the number of tests performed to identify DMRs. By filtering MRs using the criteria outlined in a forthcoming manuscript by Hagmann, Becker et al. [65], we reduced the number of MRs four fold in each species. For each identified region, pairwise statistical tests were performed for the relevant comparisons listed above. The statistical test approximates the context-specific beta binomial distribution for the region of interest. Individual and joint distributions are approximated for two samples being compared. The statistical test compares the individual sample distributions to the joint distribution using a log-odds ratio. This ratio is compared against a chi-squared distribution to obtain confidence values. For each identified region, samples were assigned to groups by separating the samples with statistically significant methylation. To confirm groupings, we first combined read counts from treatment combinations in the same group. With the combined data, the same statistical test as described above was performed to test for differential methylation. Groups were confirmed in this way to identify and filter potentially erroneous DMRs. After false discovery rate (FDR) correction using Storey's method [66], regions with an FDR below 0.01 were defined as differentially methylated regions (DMRs). To resolve overlapping DMRs, we retained the non-overlapping regions containing the maximum number of samples with statistically significant differential methylation. Apart from the criterion used to resolve overlapping DMRs, the methods follow those that will be described in detail in a manuscript by Hagmann, Becker et al. [65].

### Site-level conservation of methylation

We identified conserved sites using a published three-way whole genome alignment [47]. For CG sites, identical context was required while substitutions at the H positions were allowed in degenerate contexts as long as they did not mutate to G. Sites that transitioned contexts were not considered. Methylation rates for significantly methylated sites were then extracted from each species, tissue, and treatment combination for subsequent analysis.

### Identification of 1∶1∶1 orthologous gene pairs

Three-way orthologs were identified using the reciprocal-best blastp hit approach as implemented in the multiParanoid pipline (inParanoid v. 4.1, blast v. 2.2.26) [67].

### RNA sequencing

We sequenced each RNAseq library with 101 base pair single-end reads on the Illumina GAII instrument. We pooled twelve different samples in each lane. Each pool was sequenced over three lanes. The Illumina RTA software (version 1.13.48) performed image analysis and base calling.

### Processing and alignment of RNAseq reads

Reads were trimmed using the shore import function in SHORE version 0.9.0 [68]. Command line arguments can be found in Text S1. This function simultaneously trims reads and separates samples by barcode. Since all samples were sequenced over three lanes, after lanes are de-multiplexed sample reads were combined. Due to variable annotation qualities between species, only sequences annotated as CDS annotations were used to map RNA-seq reads. The following representative gene model annotation versions were used for each species: Crubella_183, Alyrata_107, Athaliana_167 (TAIR10) [46], [47], [50], [51]. Reads were aligned with one allowed mismatch to the appropriate annotation using bwa version 0.6.1 [69]. Read counts were obtained for each gene using a custom perl script. In summary, the script identified uniquely aligned read with a mapping quality score above 30 and stored the total read count for each target sequence. Read and alignment statistics can be found in Table S20. Raw reads are deposited at the European Nucleotide Archive under accession number PRJEB6701.

### Differential expression analysis

Differentially expressed genes were identified using the R package edgeR (3.4.2) with minor modifications [70]. Using edgeR, we estimated the dispersion parameter for each gene using estimateGLMTagwiseDisp(). Next, we fit a negative binomial generalized linear model (GLM) using glmFit(). Significance testing for differential expression was performed using a custom GLM. Significance testing in edgeR was done via term-dropping of each factor level (likelihood ratio test), and as a result performed more statistical tests than necessary. To minimize multiple testing problems, we implemented a negative binomial GLM that tested for differential expression significance using an ANOVA [71]. Dispersion estimates from edgeR were provided to the modified GLM. Using this model, differential expression analysis was performed in two ways. First, expression analysis was performed within species. There were 12 samples consisting of three replicates and four unique treatment combinations. All representative gene models were considered. The following custom GLM model was used: expression∼tissue*treatment. This included the main effects of tissue and treatment as well as their interaction. Secondly, we performed differential expression analysis between all species simultaneously. In this case, there are a total of 36 samples consisting of three replicates of each species, tissue, and treatment combination. Only 1∶1∶1 orthologous gene pairs were considered (14,395 in total). The following custom GLM model was used: expression∼species*tissue*treatment. This includes the main effects of species, tissue, and treatment as well as all two and three-way interactions. Corrections for gene length were performed, but this did not impact the results and was subsequently ignored.

### Repeat annotations

Transposon and repeat annotations for all three species were derived from the *Capsella rubella* genome paper [47], [72], [73].

## Supporting Information

Figure S1 Effects of intron insertions of transposons on gene expression. Genes with and without TEs in their introns are compared. A gene is considered expressed if it had at least 3 RPKM in three of the twelve species-specific RNA-seq samples.
(EPS)

Figure S2 Methylation rates of sequences flanking intron-inserted transposons. All cytosines in sequences flanking TEs in introns were extracted (+/−500 bp). Methylation rate for each annotated feature and context is calculated as the number of methylated cytosines over the total number of possible cytosines. Methylation rates are normalized to genome-wide methylation rates for each feature-context combination. Sites considered in our current analysis (intronic TE and +/−500 bp) were excluded from the calculation of background methylation rates. This plot also accounts for expression of the gene containing the intronic TE.
(EPS)

Figure S3 Methylation rates at genomic features of expressed genes. Genome average of methylation rates for each genomic feature. Similar to figure 2B, except annotations are only considered for genes that are expressed. A gene is considered expressed if it received at least 3 RPKM in three of the twelve species-specific RNA-seq samples. Methylation rates are normalized to the outgroup species *C. rubella*.
(EPS)

Figure S4 Genomic distribution of MRs and DMRs. A) Circos plots [74] to demonstrate the genomic distribution of MRs and DMRs in *C. rubella*, *A. lyrata*, and *A. thaliana*. Chromosome number is indicated on the inner circle. Data is plotted for 500 kb windows.
(EPS)

Figure S5 Relationship between gene body methylation and gene expression. Gene body methylation rates are plotted against either gene expression (log_2_) deciles or coefficient of variation (CV) deciles. When comparing gene body methylation with gene expression the Spearman rank correlation coefficient in *C. rubella* = 0.21, *A. lyrata* = 0.23, and *A. thaliana* = 0.24. In contrast, when comparing gene body methylation with CV the Spearman rank correlation coefficient in *C. rubella* = −0.34, *A. lyrata* = −0.19, and *A. thaliana* = −0.33.
(EPS)

Figure S6 Annotation of methylated site classes in three-way alignments. Feature annotation is shown for each methylation context. Site classes are as follows: Aligned - all C in three-way alignments. mC - methylated sites within a species. Consv. (3 species) - sites that are methylated in all three species. Gain - sites that are methylated in a single species. Loss - sites that have lost methylation in a single species.
(EPS)

Figure S7 Transposon categories for aligned methylated site classes. The top 5% of windows (10 kb) for three-way conserved sites, gains, and losses were identified. As a control, an equal number of random genomic windows were chosen. Shown is the number of bases annotated as a transposon category for the top 5% of windows in each site class normalized to the control annotation.
(EPS)

Figure S8 Centromere loss is not associated with methylation loss at aligned cytosines. Fraction of species-specific losses in methylation is plotted for each ortholog residing within ancestral centromere boundaries. Orthologs were categorized based on genomic position, either in or outside of ancestral centromere boundaries. Centromere boundaries were defined in *C. rubella* using repeat density (Fig. 3, 0.3 threshold). Orthologs residing in maintained ancestral centromeres (“No Loss”) were compared to orthologs residing in ancestral centromeres lost in *A. thaliana* (“Loss”).
(EPS)

Figure S9 Conserved methylated sites associated with conservation of exon length. Fraction of site categories that reside in exons with conserved lengths across all three species or exons of variable lengths.
(EPS)

Figure S10 Distribution of cytosines across exons. The density of exon methylation at aligned cytosines is shown for conserved methylated sites as well as for lineage-specific gains and losses of methylation. On top is the density of non-methylated aligned cytosines. There is no bias in location within an exon for non-methylated sites.
(EPS)

Figure S11 Differential gene expression. For each model, within species (top) and between species (bottom), the number of differentially expressed genes (absolute and as a fraction of expressed genes) is shown for each main effect and all interactions (p<0.05).
(EPS)

Figure S12 Species mC relationship of replicates. A) Principal component analysis on mC rates at aligned methylated positions. All contexts are considered (see Fig. 6B,C and Table S10 for further description of mC sites). Unlike figure 7, this plot considers the mC rate of each replicate at all aligned methylated positions.
(EPS)

Figure S13 Genomic distribution of DMPs. A) Circos plots [74] to demonstrate the genomic distribution of DMPs in *C. rubella*, *A. lyrata*, and *A. thaliana*. Plots are separate for tissue specific DMPs (root and shoot) or treatment specific DMPs (23°C and 4°C). Chromosome number is indicated on the inner circle. Data is plotted for 500 kb windows.
(EPS)

Figure S14 Conservation of DMRs in the absence of sequence alignments. The total number of orthologous genes containing a DMR in one, two, or three species is shown. Location of DMR overlap is separated by genomic feature. Upstream region is considered 1 kb before the start codon. Asterisk indicates two or three-way sharing of DMRs that exceeds permutation values.
(EPS)

Table S1 References for genome size. References for the genome size (in pg and Mb) as well as the total size of the genome assembly are listed for each species. Genome size references are derived from the Kew Royal Botanic Gardens Plant DNA C-values database.
(XLSX)

Table S2 Bisulfite-sequencing coverage and alignment statistics. For each sample, the total number of sequenced reads (paired and single) is shown. Also, the total number of CG, CHG, and CHH sites covered is reported along with the average genome-wide coverage of each context.
(XLSX)

Table S3 False methylation rates by coverage bin. The incomplete bisulfite conversion rate, or false methylation rate (FMR), for each sample is shown by coverage bin. For each bin, FMR is calculated as the number of cytosines in lambda DNA that are not converted to U (T in the DNA sequence) after bisulfite treatment over the total number of converted (U/T) and unconverted (C) reads.
(XLSX)

Table S4 MR and DMR statistics by sample (A) and species (B). Mean and median length of region, total number of regions, and genomic bases covered by regions are shown. Sample statistics were calculated from the combination of biological replicates.
(XLSX)

Table S5 Genome alignment metrics. Number of bases covered in three-way whole genome alignments is shown. In addition, total number of bases in methylated site classes is shown.
(XLSX)

Table S6 MR presence at genomic features. The number of genes in each species annotation that contain a MR is shown. Upstream refers to 1 kb upstream of the start codon. The number of orthologous genes with an overlapping MR is also shown.
(XLSX)

Table S7 P values of pairwise MR overlap. To test the significance of MR co-occurrence at orthologous genes a hypergeometric test was used. Significance of each test is shown here.
(XLSX)

Table S8 Two and three-way species MR overlap. The number of orthologs that contain an MR in one, two, or three species is shown (A). Permutation analysis was performed to estimate the random occurrence of one, two, and three-way overlap (10,000 permutation tests). Maximum permutation values are shown in (B). Features where the data exceeds the maximum permutation value are indicated in (C).
(XLSX)

Table S9 Gene body methylation by context. The total numbers of genes with CG, CHG, or CHH gene body methylation are shown for all genes (A) and orthologous genes (B).
(XLSX)

Table S10 Three-way genome alignment site classes by context. Total numbers of CG, CHG, and CHH sites for each alignment site class are shown.
(XLSX)

Table S11 DMP statistics by comparison. Total numbers of DMPs in each tissue and treatment comparison are shown.
(XLSX)

Table S12 DMR statistics by comparison. Total numbers of DMRs in each tissue and treatment comparison are shown.
(XLSX)

Table S13 DMR presence at genomic features. The number of genes in each species' annotation that contain a DMR is shown. Upstream refers to 1 kb upstream of the start codon. The number of orthologous genes with an overlapping DMR is also shown.
(XLSX)

Table S14 P values of pairwise DMR overlap. To test the significance of DMR co-occurrence at orthologous genes a hypergeometric test was used. Significance of each test is shown here.
(XLSX)

Table S15 Two and three-way species DMR overlap. The number of orthologs that contain a DMR in one, two, or three species is shown (A). Permutation analysis was performed to estimate the random occurrence of one, two, and three-way overlap (10,000 permutation tests). Maximum permutation values are shown in (B). Features where the data exceeds the maximum permutation value are indicated in (C).
(XLSX)

Table S16 DMPs at genomic features. The number of genes in each species annotation that contain a DMP is shown. Upstream refers to 1 kb upstream of the start codon. The number of orthologous genes with an overlapping DMP is also shown.
(XLSX)

Table S17 DMP correlation with gene expression by feature. Spearman rank correlation coefficient was calculated between the direction of differential methylation and the appropriate log_2_ fold change. Correlation coefficients were calculated separately for tissue and treatment specific DMPs. An NA value indicates that there were too few genes in a given category. Expression values are from the intraspecific expression analysis.
(XLSX)

Table S18 DMR correlation with gene expression by feature. Spearman rank correlation coefficients when comparing the degree of differential methylation for each context (extracted from the HMM model) with the appropriate log_2_ fold change. All annotated genes overlapping a DMR were considered. Expression values are from the intraspecific expression analysis. Correlation coefficients were calculated only for tissue-specific DMRs as there are too few treatment-specific DMRs. Results are only shown for DMRs overlapping CDS, intron, and upstream sequences because too few expressed genes reside in the other categories (5′ and 3′ UTRs). Upstream refers to 1 kb upstream of the start codon.
(XLSX)

Table S19 Number of DMPs between replicates. For each species, tissue, treatment combination, differentially methylated positions were identified between biological replicates. The total number of DMPs for each comparison is listed. These positions were removed from all further analyses.
(XLSX)

Table S20 RNA-seq sequencing coverage and alignment statistics. For each sample, the total number of RNA sequencing reads is shown. Read counts are also shown for mapped reads, uniquely mapped reads, and the reads that passed a mapping quality threshold (30).
(XLSX)

Text S1 Command lines for alignments. Command lines and arguments for the processing of bisulfite reads and RNA-seq reads.
(TXT)
